# Redox Properties of Human Erythrocytes Are Adapted for Vitamin C Recycling

**DOI:** 10.3389/fphys.2021.767439

**Published:** 2021-12-06

**Authors:** Michael Eigenschink, Danylo Savran, Christoph P. Zitterer, Sebastian Granitzer, Magdalena Fritz, David M. Baron, Ernst W. Müllner, Ulrich Salzer

**Affiliations:** ^1^Center for Medical Biochemistry, Max Perutz Labs Vienna, Medical University of Vienna, Vienna, Austria; ^2^Institute of Medical Genetics, Medical University of Vienna, Vienna, Austria; ^3^Department of Anaesthesia, Intensive Care Medicine and Pain Medicine, Medical University of Vienna, Vienna, Austria

**Keywords:** ascorbic acid, dehydroascorbic acid, glutathione, MRP1, GLUT1, DCytb, vitamin C auxotrophy, evolution

## Abstract

Ascorbic acid (AA; or vitamin C) is an important physiological antioxidant and radical scavenger. Some mammalian species, including *homo sapiens*, have lost the ability to synthetize AA and depend on its nutritional uptake. Erythrocytes from AA-auxotroph mammals express high amounts of the glucose transporter GLUT1. This isoform enables rapid uptake of glucose as well as dehydroascorbate (DHA), the fully oxidized form of AA. Here, we explored the effects of DHA uptake on the redox metabolism of human erythrocytes. DHA uptake enhanced plasma membrane electron transport (PMET) activity. This process is mediated by DCytb, a membrane bound cytochrome catalyzing extracellular reduction of Fe^3+^ and ascorbate free radical (AFR), the first oxidized form of AA. DHA uptake also decreased cellular radical oxygen species (ROS) levels. Both effects were massively enhanced in the presence of physiological glucose concentrations. Reduction of DHA to AA largely depleted intracellular glutathione (GSH) and induced the efflux of its oxidized form, GSSG. GSSG efflux could be inhibited by MK-571 (*IC*_50_ = 5 μM), indicating involvement of multidrug resistance associated protein (MRP1/4). DHA-dependent GSH depletion and GSSG efflux were completely rescued in the presence of 5 mM glucose and, partially, by 2-deoxy-glucose (2-DG), respectively. These findings indicate that human erythrocytes are physiologically adapted to recycle AA both intracellularly *via* GLUT1-mediated DHA uptake and reduction and extracellularly *via* DCytb-mediated AFR reduction. We discuss the possibility that this improved erythrocyte-mediated AA recycling was a prerequisite for the emergence of AA auxotrophy which independently occurred at least twice during mammalian evolution.

## Introduction

Ascorbic acid (AA), commonly known as vitamin C, is an antioxidant and radical scavenger. AA preferably engages in one-electron transfer-reactions resulting in the formation of ascorbate free radicals (AFR) but can also be further oxidized to dehydrascorbic acid (DHA) upon losing a second electron. Two molecules of AFR can disproportionate to DHA and AA. An additional characteristic of AFR is its high reactivity with radicals and poor reactivity toward non-radical species (Tu et al., 2017). Due to these properties, AA and AFR are important physiological antioxidants for systemic radical scavenging. AA biosynthesis predominantly takes place in the liver of most mammalian species (Chatterjee et al., 1961). However, some species (fruit bats, guinea pigs, and higher primates including *homo sapiens*) depend on nutritional AA uptake since changes in the L-gulono-γ-lactone oxidase (GLO) gene inactivated the last step in AA biosynthesis (Chatterjee, 1973). Interestingly, rats with defects in AA biosynthesis require higher nutritional AA uptake than the “natural” AA auxotroph guinea pigs to prevent adverse systemic effects (Horio et al., 1985). This indicates that adaptations must have occurred during the evolution of AA auxotroph organisms to minimize systemic loss of vitamin C. Erythrocytes of all mammalian species that depend on dietary vitamin C supply express the glucose transporter isoform GLUT1 whereas species capable of AA biosynthesis express other GLUT isoforms in the plasma membrane of their erythrocytes (Montel-Hagen et al., 2008). In contrast to other isoforms, GLUT1 and 3 also efficiently facilitate the transport of DHA (Rumsey et al., 1997; Tu et al., 2015). The joint emergence of the loss of GLO activity and expression of GLUT1 in erythrocytes occurred independently at least twice during mammalian evolution (Drouin et al., 2011), suggesting that (i) this isoform switch is essential for vitamin C auxotroph species and (ii) erythrocytes play an important role in redox processes involving vitamin C. In contrast to AFR, DHA is unstable and quickly degraded to 2,3-diketo-1-gulonic acid in an irreversible reaction (Winkler, 1987). Hence, rapid recycling of DHA into the reduced AA state is crucial for vitamin C auxotrophs to minimize systemic loss of this vitamin. This is likely to be an essential evolutionary adaptation to reduce irreversible degradation of DHA resulting from oxidative processes within the blood stream.

Due to their function as oxygen carriers, erythrocytes contain several enzymes protecting against damage by radical oxygen species (ROS) as well as high concentrations of the antioxidant glutathione (GSH), ranging from 0.4 to 3.0 mM (van ’t Erve et al., 2013). Normally, the ratio between reduced GSH and its oxidized form, GSSG, is about 7:1. Erythrocytes are equipped with enzymes for GSH synthesis and have high-efficiency importers for its amino acid components (Raftos et al., 2010). GSH reacts with superoxides directly, is involved in degradation of hydrogen peroxide and lipid peroxides *via* glutathione peroxidases, and in covalent modifications of toxic xenobiotics by glutathione S-transferases (GSTs) (Ayala et al., 2014). By far the most abundant GST variant in human erythrocytes is GSTO-1 (Bryk and Wisniewski, 2017), the isoform 1 of the omega class of GSTs. It is an enzyme with specific DHA reductase activity (Zhou et al., 2012). Apart from a GSTO-1-mediated process, DHA can also be directly reduced by GSH, generating AA and GSSG (Winkler, 1992). Thus, with their high content of GLUT1, GSH and GSTO-1 human erythrocytes are well equipped for high-capacity uptake of DHA and its fast regeneration into the stable AA state.

*Trans-*membrane electron transport at the inner mitochondrial membrane is essential for oxygen-dependent transformation of nutrient-derived reduction equivalents into ATP-stored chemical energy. *Trans-*plasma membrane electron transfer (PMET), in contrast, is more ambiguously defined as a process where reduction equivalents, either electrons or reductants, are exported to the extracellular environment. PMET is likely involved in a number of physiological processes (Lane and Lawen, 2008) and seems to play a crucial role in redox homeostasis of tumor cells (Sherman et al., 2019). In erythrocytes, various approaches have been undertaken to study *trans-*plasma membrane electron/reductant transport, and several mechanisms have been discussed (Kennett and Kuchel, 2003). These processes have therefore been differently referred to as plasma membrane electron transfer (PMET) (Kennett and Kuchel, 2003), plasma membrane redox system (PMRS) (Rizvi et al., 2006), or (extracellular) ascorbate recycling (Mendiratta et al., 1998). In most studies, transmembrane flow of electrons/reductants was assessed by incubating erythrocytes with the electron acceptor [Fe(CN)_6_]^3–^ (ferricyanide) and the amount of [Fe(CN)_6_]^4–^ (ferrocyanide) generated was quantified by colorimetric assays. Human erythrocytes express the duodenal isoform of cytochrome b561 (DCytb) (Su et al., 2006). DCytb is a transmembrane protein with two heme groups, known to be involved in duodenal iron absorption by reducing dietary Fe^3+^ prior to its uptake by enterocytes (McKie et al., 2001). DCytb has ascorbate binding sites, both at the cytoplasmic and the apical side, indicating that it not only reduces Fe^3+^ but also regenerates ascorbate from ascorbate free radicals (AFR) at the apical binding site, using electrons provided by ascorbate at the cytoplasmic binding site (Ganasen et al., 2018). Its presence at the erythrocyte plasma membrane indicates that the main function of DCytb in these cells is extracellular ascorbate recycling (VanDuijn et al., 2000). The AA-dependent export of electrons during extracellular ascorbate recycling (which can be regarded as PMET activity), however, requires an efficient mechanism of intracellular AFR reduction mediated by thioredoxin reductase, using NADPH as a reductant (May et al., 1998).

DHA uptake into erythrocytes, its recycling to AA within erythrocytes, and PMET activity are processes that consume intracellular reduction equivalents. This study investigates the effects of DHA uptake into human erythrocytes with respect to PMET activity and changes in intracellular ROS as well as GSH levels. Furthermore, we explore the consequences of DHA-dependent GSSG accumulation. In order to evaluate the physiological relevance of our findings, these effects were also studied in the absence and presence of increasing amounts of glucose and 2-deoxy-glucose (2-DG).

## Materials and Methods

### Sample Collection and Study Population

This study was approved by the ethics commission of the Medical University of Vienna (EK Nr. 1752/2020). Blood was taken from 10 healthy volunteers between 18 and 30 years of age, with a BMI between 19 and 30 kg/m^2^. Exclusion criteria were: pre-existing health conditions, regular smoking, substance abuse, regular usage of medications and/or vitamin supplements. Participants were specifically asked to remain abstinent from alcohol and citrus fruits for 3 days, avoid excessive physical activity for at least 2 days, and fast for at least 8 h prior to blood sampling.

Blood was collected into 9 mL EDTA vacutainers (Greiner Bio-One, Kremsmünster, Austria) from an antecubital vein. Freshly drawn blood was aliquoted into 2 mL microcentrifuge tubes (Eppendorf, Hamburg, Germany), immediately centrifuged for 1 min at 8000 *g* (Eppendorf, 5415C), and plasma and buffy coat discarded. Subsequently, cells were resuspended in PBS, washed three times, and diluted to a suspension of 4 × 10^6^ cells/μL.

### Cell Counting

Aliquots of erythrocyte suspensions were diluted 10 or 40 times in PBS. For cell counting and quality control, samples were further diluted 1000 times in CASYton (Roche Applied Science, Penzberg, Germany) and their absolute number, diameter and volume determined using a CASY^*TM*^ cell counter (Roche Applied Science) employing a 60 μl capillary. Samples with aberrant peak shape or cell volume were excluded from further analyses. All measurements were performed in duplicates.

### Plasma-Membrane Electron Transfer Assay

2 × 10^6^ RBC/μL were incubated with 2 mM DHA (Sigma, 261556, Burlington, NJ, United States) or PBS in the absence or presence of 5 mM glucose (AppliChem, A3666, Darmstadt, Germany) for 15 min at room temperature (RT). Erythrocytes were then washed three times and resuspended in PBS. Afterward, cells were resuspended in 1 mM [Fe(CN)_6_]^3–^ (AppliChem, A3883) with or without 5 mM glucose at a concentration of 5 × 10^5^ cells/μL. At given time intervals, aliquots from samples were centrifuged for 1 min at 8000 *g* and supernatants recovered. Supernatants were then transferred into semi-micro cuvettes (Sarstedt, Nümbrecht, Germany) and diluted in PBS. Afterward, samples were incubated with a freshly prepared master mix consisting of bathophenanthroline-disulfonic acid (6 mM) (Sigma, 146617), sodium citrate (0.2 M) (Merck, 1.06432), sodium acetate (3 M, *pH* = 6.5) (AppliChem, 131632), and FeCl_3_ (3 mM) (Merck, 236489) in the dark for 25 min as described by Avron and Shavit (1963). Subsequently, extinction was measured at 540 nm (U-2000 spectrophotometer, Hitachi, Tokyo, Japan). Ferrocyanide (Sigma, P3289) standard curves were determined and experimental data fitted to respective values.

### Determination of Intracellular Radical Oxygen Species Levels by Flow Cytometry

RBCs were suspended to a final concentration of 1 × 10^4^ cells/μL in PBS and incubated with 5 μM 2′,7′-dichlorodihydrofluorescein-diacetate (H_2_DCF-DA) (Sigma, D6883) for 30 min. H_2_DCF-DA is a colorless reagent that becomes partially trapped in cells upon deacetylation and reacts with ROS to form the fluorescent DCF (Chen et al., 2010). Erythrocytes were then centrifuged for 4 min at 30 g and supernatants discarded. Resuspended cells were incubated at DHA concentrations from 0.1 to 2 mM in the presence or absence of 5 mM glucose. After 15 min, samples were collected and ROS production measured by flow cytometry (LSRFortessa^*TM*^ Cell Analyzer, BD Biosciences, Franklin Lakes, NJ, United States). All procedures were performed in the dark at RT.

### Determination of Intracellular Thiol Content by Flow Cytometry

To assess the loss of intracellular glutathione (GSH) upon DHA uptake, erythrocytes at 2 × 10^6^ cells/μL were incubated at DHA concentrations from 0.1 to 2 mM for 15 min at RT. Afterward, samples were washed three times in PBS and resuspended at 1 × 10^4^ cells/μL. For GSH regeneration assays, 2 × 10^6^ RBC/μL were incubated with 2 mM DHA in the presence of either glucose or 2-deoxy-glucose (2-DG) (Sigma, D8375) at concentrations between 5 μM and 5 mM. After 15 min, samples were quickly diluted in PBS to a final concentration of 1 × 10^4^ cells/μL. Incubations with 3 mM of 1-chloro-2,4-dinitrobenzene (CDNB) (Sigma, 138630) for 30 min at RT were used as negative controls for both types of experiments. CDNB is a substrate of GSH S-transferase ρ. Incubation of erythrocytes with 3 mM CDNB for 30 min has been shown to specifically deplete 96% of intracellular GSH by conversion to 2,4-dinitrophenyl-S-glutathione (Awasthi et al., 1981). Cells were then incubated with 50 μM monobromobimane (MBB) (Sigma, B4380) for 10 min in the dark (Cossarizza et al., 2009). MBB spontaneously reacts with thiols in a biphasic reaction, preferring GSH over protein-sulfhydryls (Hedley and Chow, 1994). Suspensions were centrifuged at 4°C at 30 *g* for 4 min and the supernatant was discarded. Cells were resuspended in PBS and analyzed by flow cytometry (LSRFortessa^*TM*^ Cell Analyzer, BD Biosciences).

### Enzymatic Determination of Oxidized Glutathione (GSSG)

2 × 10^6^ RBC/μL were incubated with PBS or 2 mM DHA in the presence or absence of 5 mM glucose or PBS with 100 μM of the multidrug resistance protein (MRP) 1/4 inhibitor MK-571 (Sigma, M7571) for 15 min at RT. Erythrocytes were then washed three times with PBS or PBS containing 5 mM glucose, respectively, and resuspended in either PBS, PBS containing 5 mM glucose or PBS containing 100 μM MK-571. Aliquots were collected at various time points, centrifuged at 8000 *g* for 1 min and supernatants recovered. To obtain DHA, glucose, and 2-DG dose-response curves, erythrocytes were incubated with the respective compounds as described in the section on flow cytometry of intracellular thiols. After 90 min, samples were centrifuged for 1 min at 8000 *g* and supernatants recovered. For MK-571 dose-response measurements, erythrocytes were incubated with 2 mM DHA and inhibitor concentrations from 0.1 to 100 μM for 15 min at RT and processed as stated above. Based on protocols by Rahman et al. (2006) and Giustarini et al. (2013), a master mix containing 1.66 units/mL GSH reductase (Sigma, G3664) and 0.84 mM 5,5′-dithiobis-2-nitro benzoic acid (DTNB) (Sigma, D8130) was prepared freshly. Supernatants were diluted sevenfold in PB200 (0.16 mM Na_2_HPO_4_, 0.038 mM KH_2_PO_4_, *pH* = 7.4), and 120 μl master mix was added. After 30 sec to allow for conversion of GSSG to GSH, NADPH (Roche Diagnostics, 10107824001) was added to a final concentration of 54.54 μM and sample kinetics measured after 15 sec of equilibration continuously for 4 min at 412 nm (U-2000 spectrophotometer, Hitachi). In addition, GSSG standard-curves consisting of freshly prepared GSSG (Sigma, G4376) solutions (1.25, 2.5, 5, 10 μM) and a 10 μM GSSG frozen standard control were established with each experiment. To obtain reliable GSSG-efflux estimates, linear regression was applied to standard curves after correction for standard controls and sample data fitted to the respective regressions.

### Statistical Analyses

All statistical analyses were performed in GraphPad Prism 9.1.1 (GraphPad Software Inc., San Diego, CA, United States). Dose-response curves were approximated by non-linear regression. Longitudinal data were analyzed in a mixed model. The Šidák correction was used as a rather conservative estimate to control for multiple comparisons (Liu et al., 2010). Where applicable, results of mixed models are presented as mean differences with confidence intervals. *P*-values are indicated as asterisks in bar charts, respectively. Data are shown as mean values with standard deviations. Flow cytometry data were exported as FCS 3.1 files and visualized as histograms with FlowJo^*TM*^ Software Version 10 (Becton, Dickinson and Company, 2019). Time series were additionally analyzed by linear regression to retrieve respective equations.

## Results

**FIGURE 1:**
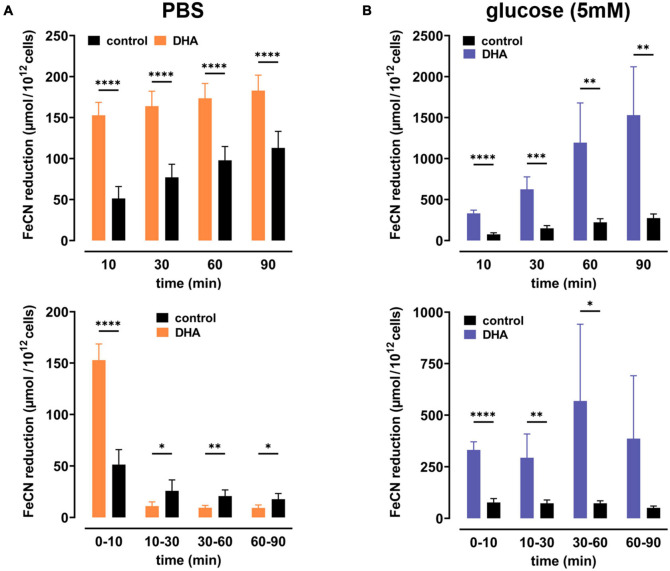
Dehydroascorbic acid (DHA) uptake and glucose synergistically fuel erythrocyte plasma membrane electron transport. Erythrocytes (*n* = 8) were pre-incubated in 2 mM DHA or PBS (=control, black) in the presence (blue) or absence (orange) of glucose. After washing, cells were suspended in 1 mM ferricyanide in PBS **(A)** or PBS containing 5 mM glucose **(B)**. Aliquots were removed at indicated time points and the amount of ferrocyanide in supernatants assessed as described in section “Materials and Methods.” Mean values of reduced ferrocyanide are given in μmol/10^12^ cells and shown both as cumulative values (upper panels) and amount generated between time points (lower panels), respectively. Please mind the different scales of the y-axes in panels **(A,B)**.

To study dehydroascorbate (DHA)-dependent changes in erythrocyte redox properties we first investigated erythrocyte plasma membrane electron transport (PMET) activity by quantifying the amount of extracellular ferricyanide reduction over time. Upon pre-incubation with 2 mM DHA, ferrocyanide concentrations increased trifold [153 ± 16 μmol/10^12^ cells; mean difference (MD): 102 μmol, confidence interval (CI): (78–125 μmol)] within 10 min as compared to controls (51 ± 15 μmol/10^12^ cells) (Figure 1A). After this initial spike in electron export, however, PMET activity of DHA-loaded erythrocytes strongly decreased over time to a level even below that of controls. In the presence of 5 mM glucose, PMET increased both in DHA-loaded and control erythrocytes (Figure 1B). Remarkably, this increase in PMET activity was robust, persisting at a high level in DHA-loaded cells for up to 90 min (1530 ± 590 μmol/10^12^ cells). Thus, upon DHA uptake erythrocytes supplied with physiological glucose concentrations reveal their high and long-lasting capacity to export electrons/reduction equivalents to extracellular acceptors.

**FIGURE 2:**
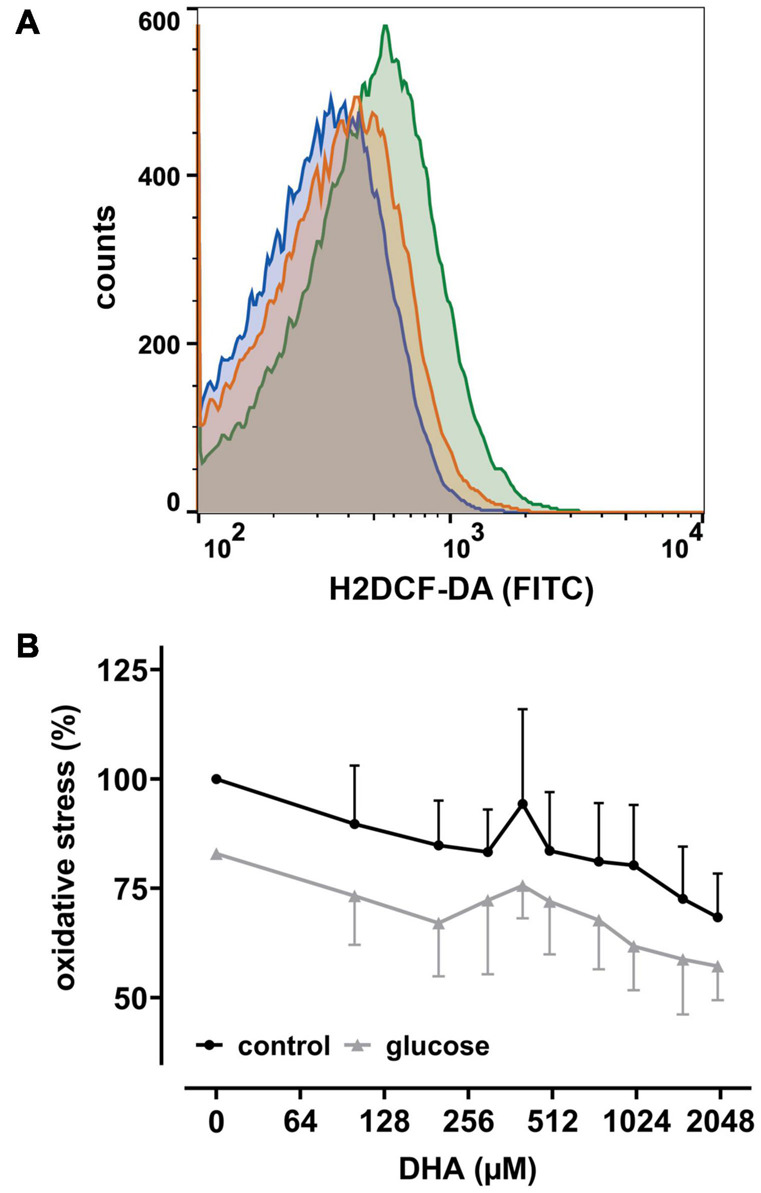
DHA and glucose lower intracellular levels of reactive oxygen species (ROS). **(A)** Histogram depicting quantitative changes of ROS in erythrocytes treated with 2 mM DHA in the presence (blue) or absence (orange) of 5 mM glucose or PBS only (green). **(B)** Erythrocytes (*n* = 6) loaded with 2′,7′-dichlorodihydrofluorescein-diacetate (H2DCF-DA) were treated with various amounts of DHA for 15 min in the presence (gray) or absence (black) of 5 mM glucose at RT and processed for flow cytometry. Mean fluorescence intensity (MFI), reflecting intracellular ROS, is given in percent after normalization to the MFI of PBS control cells (set to 100%).

Since uptake of DHA and its reduction increases intracellular AA (May et al., 2001), we next asked whether this affects intracellular levels of reactive oxygen species (ROS) in erythrocytes. Therefore, cells were labeled with the cell-permeant reagent 2′,7′-dichlorodihydrofluorescein-diacetate (H2DCF-DA), a widely used ROS indicator. Afterward samples were incubated with increasing amounts of DHA and analyzed by flow cytometry. Intracellular ROS levels indeed decreased with increasing DHA concentrations during pre-incubation (Figure 2). Erythrocytes treated with 2 mM DHA had intracellular ROS levels of 68 ± 10% in comparison to controls. Physiological concentrations of glucose reduced intracellular ROS levels from 100% to 83 ± 13% in the absence and 83% to 57 ± 8.0% in the presence of 2 mM DHA, i.e., the effects of DHA and glucose on intracellular ROS levels were additive (Figure 2B).

**FIGURE 3:**
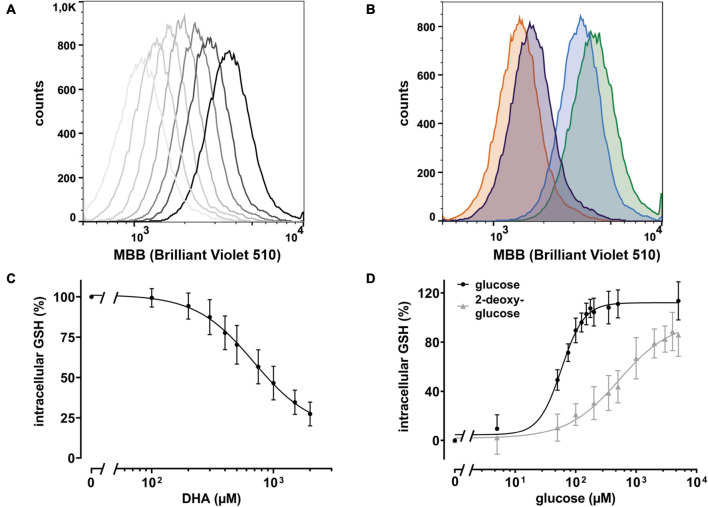
DHA-dependent depletion of cellular glutathione (GSH) is rescued by glucose and 2 deoxy-glucose (2-DG). **(A)** Histogram of intracellular GSH after incubation with DHA (0.3–2.0 mM, dark to light gray). The black line indicates PBS control cells. **(B)** Histogram of dose dependent rescue of intracellular GSH upon incubation with 2 mM DHA (orange) in the presence of 100 μM glucose (blue) or 100 μM 2-DG (violet) versus PBS control cells (green). **(C)** Erythrocytes (*n* = 11) were treated with various amounts of DHA for 15 min. Cells were then washed and incubated with the fluorescent thiol reagent monobromobimane (MBB) for 10 min. Afterward cells were centrifuged, resuspended in PBS, and processed for flow cytometry. Cells treated for 30 min with 3 mM 1-chloro-2,4-dinitrobenzene (CDNB), a glutathione-S-transferase ρ substrate capable of depleting 96% of cellular GSH within 30 min, were used as negative, cells in PBS as positive controls. MFI values of DHA-treated samples are given as percentages. Data were normalized to the MFI of positive (100%) and negative controls (0%), respectively. **(D)** Erythrocytes (*n* = 11) were treated with 2 mM DHA in the presence of indicated concentrations of glucose or 2-DG for 15 min at RT, diluted, and incubated with the fluorescent thiol reagent MBB for 10 min. Afterward, cells were centrifuged, resuspended in PBS, and analyzed by flow cytometry. MFI values are given as percentages after normalizing the data to the MFI of cells pretreated with PBS (100%) or 2 mM DHA without glucose or 2-DG (0%), respectively. Mind that cells treated with 2 mM DHA in the presence of >125 μM glucose have higher intracellular GSH levels than cells treated with PBS only.

Intracellular regeneration of AA upon DHA uptake requires 2 moles of reduction equivalents per mole of DHA. Since the major intracellular antioxidant GSH is highly abundant in erythrocytes and known to reduce DHA both directly and in an enzyme-mediated manner (Winkler, 1992; Zhou et al., 2012), we asked how DHA loading affects erythrocyte GSH levels. Assuming that GSH is the predominant low molecular weight thiol in erythrocytes, we used monobromobimane (MBB), a dye that becomes fluorescent upon reaction with such molecules, and flow cytometry to estimate changes in the GSH levels of erythrocytes upon DHA loading. There was an inverse correlation between DHA concentrations during pre-incubation and intracellular GSH content (Figure 3). At 2 mM DHA, intracellular GSH was reduced to 27 ± 7% as compared to that of cells pre-treated with PBS only (Figure 3C). Depletion of cellular GSH to 50% was achieved by pre-incubation with about 900 μM DHA. Since glutathione reductase, an enzyme highly abundant in erythrocytes, regenerates GSH in a NADPH-dependent process, we addressed the question whether glucose could rescue DHA-induced loss of GSH. In fact, erythrocytes loaded with 2 mM DHA in the presence of 5 mM glucose had GSH levels of 114 ± 16% as compared to cells pretreated with PBS only. The minimal glucose concentration for full recovery was 150 μM and half-maximal recovery occurred at 58 μM glucose [CI: (53–64 μM); *EC*_50_, relative] (Figure 3D). Interestingly, 2-deoxy-glucose (2-DG), a glucose derivative inhibiting glycolysis (Pajak et al., 2019), could also rescue DHA-dependent GSH depletion [*EC*_50_ = 578 μM, relative; CI: (386–1595 μM)], however, not to full extent even at 5 mM (86 ± 17% GSH recovery) (Figure 3D).

**FIGURE 4:**
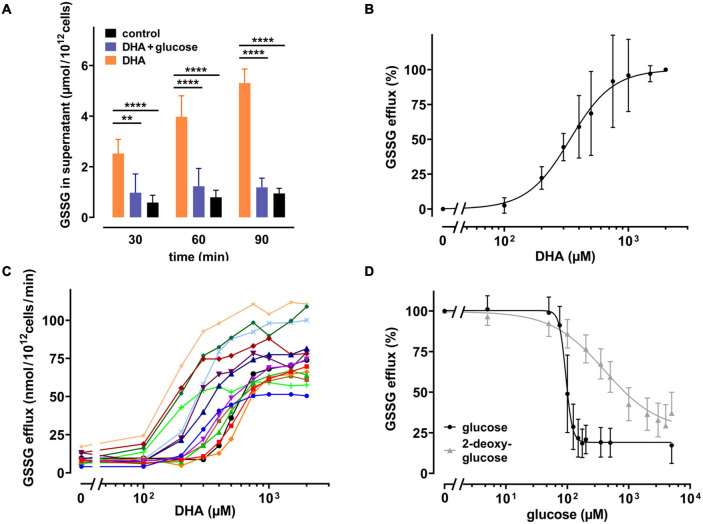
DHA-dependent GSSG efflux from erythrocytes is rescued by glucose as well as 2-DG. **(A)** Erythrocytes (*n* = 7) were incubated with 2 mM DHA in the absence (orange) or presence (blue) of 5 mM glucose for 15 min at RT. Control incubations were carried out in PBS (black). Afterward, cells were washed and resuspended in PBS (black and orange) or PBS containing 5 mM glucose (blue). Aliquots were taken at indicated time points and GSSG concentrations in the supernatant enzymatically assessed as described in the section “Materials and Methods”. **(B,C)** Erythrocytes (*n* = 14) were treated with various amounts of DHA for 15 min at RT, washed and resuspended in PBS for 90 min. GSSG content in the supernatants was assessed and the efflux rates calculated. Data are given as mean values in percent after normalizing to control conditions (pre-treatment with 2 mM DHA in PBS - 100%; PBS control cells - 0%, respectively) **(B)**, or in absolute values for individual experiments **(C)**. **(D)** Erythrocytes (*n* = 14) were treated with 2 mM DHA in the presence of glucose (black) or 2-DG (gray) for 90 min at RT and the GSSG content in the supernatant assessed. GSSG efflux rates are given in percent after normalization to control conditions (pre-treatment with 2 mM DHA in PBS without glucose or 2-DG - 100%; PBS control cells - 0%, respectively).

It is known that human erythrocytes release GSSG in an ATP-dependent manner when exposed to hydrogen peroxide (Srivastava and Beutler, 1969). Thus, it was evaluated whether the DHA-dependent decrease in the GSH/GSSG ratio would also trigger GSSG efflux. We therefore incubated erythrocytes with 2 mM DHA for 15 min, removed it by washing, and continued the incubation for another 90 min in the presence or absence of glucose. Indeed, GSSG in the supernatant increased over time for DHA-loaded erythrocyte samples (5.3 ± 0.55 μmol/10^12^ cells after 90 min) (Figure 4A). In contrast, efflux was reduced to baseline levels upon incubation with 5 mM glucose. GSSG efflux correlated with the dose of DHA during pre-loading, was nearly saturated at about 1000 μM DHA (96 ± 26%) and half-maximal at 338 μM [CI: (309–368 μM); *IC*_50_, absolute] (Figure 4B). Interestingly, maximal GSSG efflux rates from erythrocytes considerably varied between donors (77 ± 18 nmol/10^12^ cells/min at 2 mM DHA) (Figure 4C), probably due to inherent variations in erythrocyte GSH levels (van ’t Erve et al., 2013). We further tested the sensitivity of the glucose-dependent inhibition of GSSG efflux and compared it to the effect of 2-DG. GSSG efflux was half-maximal at 95 μM [CI: (90–99 μM); *IC*_50_, relative] glucose and maximal inhibition was achieved at about 175 μM glucose (19 ± 9 μM) (Figure 4D). 2-DG also reduced GSSG efflux, but required higher concentrations as compared to glucose. A 50% inhibition of GSSG efflux was reached at 376 μM [CI: (309–477.3 μM); *IC*_50_, relative] 2-DG, and 5 mM 2-DG reduced GSSG efflux to only 37 ± 13%.

**FIGURE 5:**
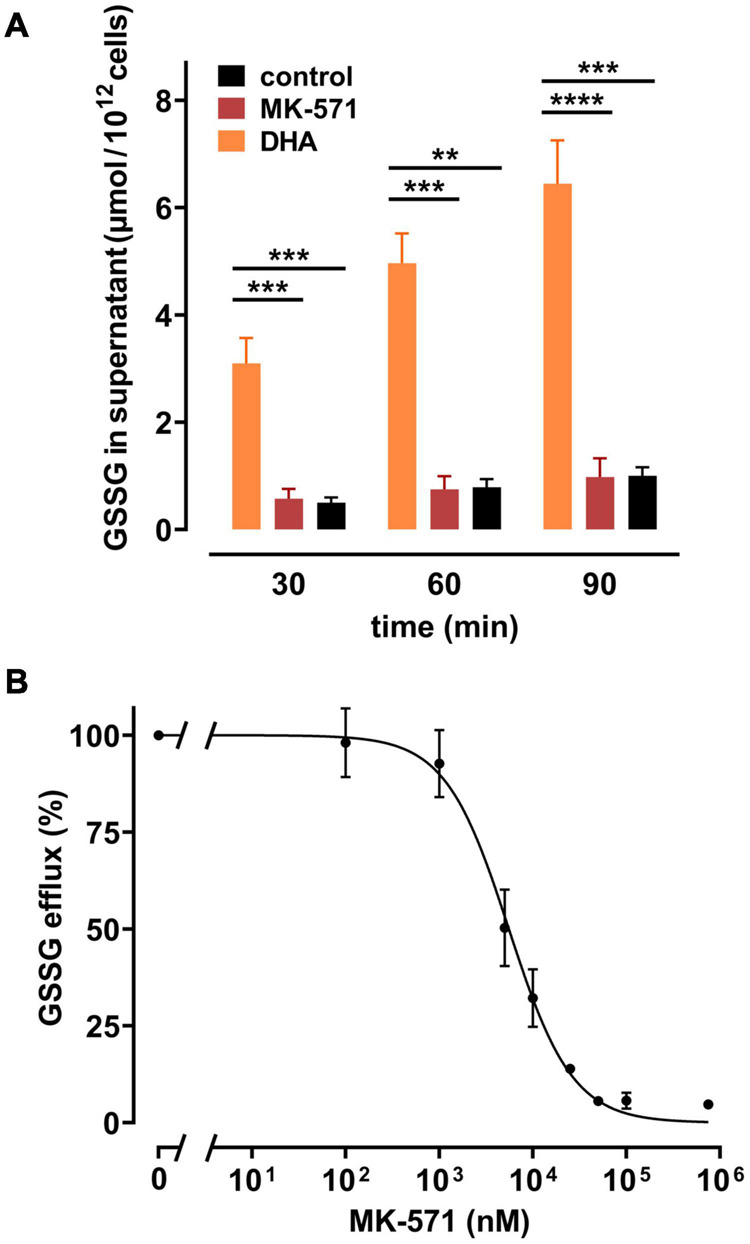
Multidrug resistance protein (MRP) 1/4 inhibitor MK-571 blunts DHA-induced GSSG efflux. **(A)** Erythrocytes (*n* = 5) were incubated with 2 mM DHA in the absence (orange) or presence (red) of 100 μM of the multidrug resistance protein (MRP1/4) inhibitor MK-571 for 15 min at RT. Control incubations were performed in PBS (black). Cells were then washed and further incubated in PBS (black and orange) or PBS containing 100 μM MK-571 (red). Abundance of GSSG in the supernatant was assessed at indicated timepoints by an enzymatic assay, as described in the section “Materials and Methods”. **(B)** Erythrocytes (*n* = 5) were treated with 2 mM DHA and various concentrations of MK-571 for 15 min at RT. After incubation, cells were washed and further incubated in PBS containing the respective concentrations of MK-571 for 90 min. GSSG efflux data were normalized to respective controls (pre-treatment with 2 mM DHA in PBS - 100%; PBS control cells - 0%, respectively).

Release of GSSG/GSH from peroxide-treated or *Plasmodium falciparum*-infected erythrocytes can be inhibited by MK-571, indicating the involvement of the multidrug resistance associated protein 1 (MRP1) in GSSG efflux (Barrand et al., 2012; Ellison and Richie, 2012). We therefore tested whether DHA-induced GSSG efflux was also mediated by this transporter. Erythrocytes were treated with MK-571 during DHA loading and tested for release of GSSG. 100 μM MK-571 drastically reduced GSSG efflux from erythrocytes pre-loaded with 2 mM DHA to the level of control samples (Figure 5A). The *IC*_50_ (absolute) for MK-571 to inhibit DHA-dependent GSSG release was 5.5 μM [CI: (4.8–6.2 μM)] (Figure 5B). Control PMET assays of MK-571-treated cells excluded the possibility that DHA-uptake was impaired in the presence of MK-571 (data not shown).

**FIGURE 6:**
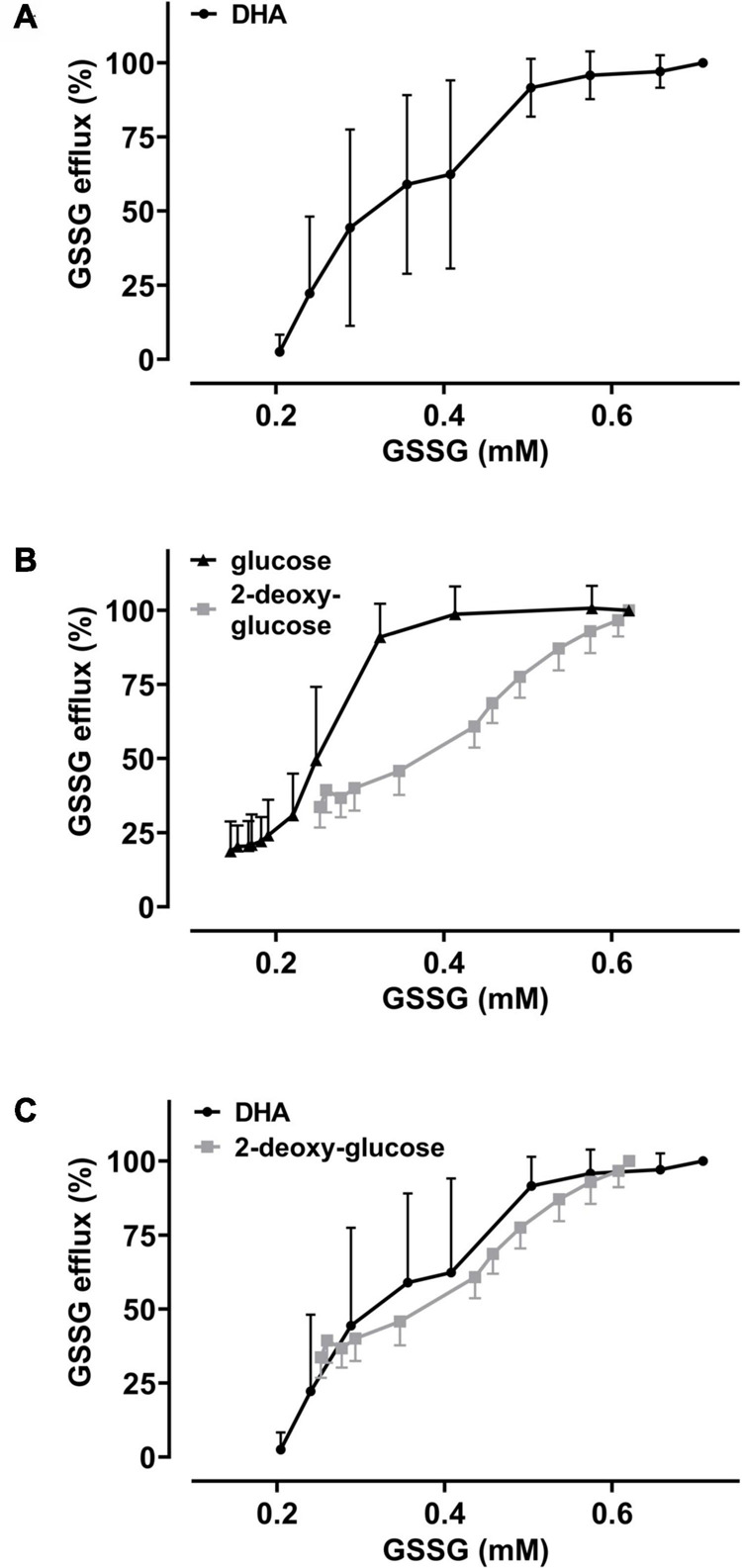
The metabolic state of erythrocytes governs the dependency of GSSG efflux on intracellular GSSG content. Amount of cellular GSSG efflux in relation to cellular GSSG content upon DHA treatment in the absence and presence of glucose or 2-DG, respectively. Values were calculated from flow cytometry GSH depletion data (Figure 3) and as described in the section “Results.” Data of GSSG efflux upon DHA treatment (± glucose or 2-DG, respectively) are derived from Figure 4. Graphs combine these data to highlight the correlation of GSSG efflux with cellular GSSG content under various conditions. **(A)** Dependency of GSSG efflux on cellular GSSG levels upon pre-treatment of cells with various amounts of DHA (data from Figures 3C, 4B). **(B)** Dependency of GSSG efflux on cellular GSSG levels upon pre-treatment of cells with 2 mM DHA and various amounts of glucose (black) or 2-DG (gray) (data from Figures 3D, 4D). **(C)** Overlay of DHA (black) data from panel **(A)** with 2-DG (gray) data from panel **(B)**.

The GSH depletion data can be merged into a numerical estimation of intracellular GSSG levels based on the following approximations: (i) DHA-dependent loss of GSH can be coupled to the formation of GSSG at a ratio of 2:1; (ii) the DHA-dependent loss of cellular GSH was estimated by normalizing to cells incubated in PBS only (set to 100%) and cells treated with 1-chloro-2,4-dinitrobenzene (CDNB) set to 0%, assuming total depletion of free GSH under these conditions (Figure 3C); (iii) mean GSH and GSSG content in human erythrocytes should be 1.4 mM and 0.214 mM, respectively, based on published data (van ’t Erve et al., 2013). Half-maximal GSSG efflux was obtained in erythrocytes upon preloading with about 350 μM DHA (Figure 4B). This DHA concentration induced a GSH depletion of about 20% (Figure 3C) which translates into a change of intracellular GSH to 1.12 mM and GSSG to 0.35 mM, respectively (0.21 mM basal plus 0.14 mM DHA-induced GSSG). The corresponding correlation curve between cellular GSSG content and GSSG efflux based on these considerations is shown in Figure 6A for each concentration of DHA. Another correlation can be plotted for the metabolic rescue of DHA-dependent GSH depletion by increasing concentrations of glucose and 2-DG (Figure 6B). In the presence of glucose, near maximal GSSG efflux was already reached at about 350 μM GSSG, whereas GSSG levels had to exceed 600 μM to achieve maximal efflux when cells were supplemented with 2-DG. Interestingly, the correlation curves of 2-DG and DHA almost overlap, suggesting comparable metabolic conditions in these two experimental settings (Figure 6C).

## Discussion

In this study we show that uptake of DHA largely affects the redox metabolism of human erythrocytes. DHA reduction and the concomitant increase in intracellular AA lowers cellular ROS levels and elevates PMET activity of the cells. On the other hand, DHA reduction is associated with depletion of intracellular GSH and export of GSSG *via* the ATP binding cassette transporter MRP1/4. Importantly, in the presence of physiological glucose concentrations, PMET activity is further increased whereas intracellular GSH depletion and GSSG export are blunted. In the light of pertinent phylogenetic findings, these results suggest that erythrocytes of the vitamin C auxotroph species *homo sapiens* are evolutionary adapted to maximize dietary uptake of vitamin C and minimize its loss in the blood stream.

In the absence of glucose, DHA loading resulted in an MK-571-inhibitable efflux of GSSG from erythrocytes (Figure 5). MRP1-mediated GSSG efflux was previously reported to be triggered by exposure to oxidative stress and upon infection with *Plasmodium falciparum* (Barrand et al., 2012; Ellison and Richie, 2012). The presence of MRP1 in erythrocytes, its activity, substrate specificity, and inhibition have already been described before (Dekkers et al., 1998; Mrowczynska et al., 2005; Wu et al., 2005). Recent proteomic analyses revealed that three isoforms of the MRP type of ATP binding cassette (ABC) transporters, MRP1, MRP4, and MRP5, are present at the erythrocyte membrane (Bryk and Wisniewski, 2017). MK-571 inhibits MRP1 and MRP4 with *IC*_50_ values of 1.1 and 0.41 μM, respectively, as assessed on human erythrocyte inside-out vesicles (Wu et al., 2005). The *IC*_50_ of MK-571 for MRP5 is 40 μM, as determined in a different cellular system (Reid et al., 2003). We found the half-maximal inhibitory concentration of MK-571 for DHA-induced GSSG efflux to be about 5 μM (Figure 5B). This intermediary value could indicate the involvement of all three isoforms in this process. Alternatively, higher inhibitory concentrations could be necessary in the normal right side-out as compared to the artificial inside-out situation. Therefore, exclusive involvement of MRP1 and/or MRP4 in DHA-induced GSSG efflux cannot be excluded and has yet to be addressed in detail. It can be roughly approximated that GSSG efflux from DHA loaded cells is half-maximal at intracellular GSSG concentrations of 315 μM (Figure 6A). For MRP1 expressed in membrane vesicles from HeLa T5 cells, the *K*_*m*_ value for ATP-dependent transport of GSSG was 93 ± 26 μM (Leier et al., 1996). Considering that our efflux data were obtained in the absence of glucose, diminished ATP levels could reduce the activity of the ATP-dependent GSSG transporter, thus accounting for the higher concentration of 315 μM GSSG required for half-maximal efflux. In fact, increasing glucose concentrations not only gradually diminished intracellular GSSG levels upon pre-treatment with 2 mM DHA but concomitantly decreased intracellular concentration of GSSG required for half-maximal efflux to 245 μM (Figure 6B). Thus, glucose not only provides reduction equivalents (NADPH) for GSH regeneration but also ATP for earlier onset of maximal GSSG efflux by MRP isoforms.

Metabolic rescue of DHA-dependent GSH depletion by glucose and 2-DG revealed interesting differences. 5 mM glucose increased intracellular GSH levels of DHA treated cells to 114% as compared to non-treated cells in the absence of glucose (Figure 3D). This indicates that reduction equivalents derived from active glucose catabolism more than fully compensate the oxidation equivalents generated during DHA uptake and subsequent AA regeneration. In contrast, 5 mM 2-DG is not fully capable to counter the loss of reduction equivalents upon DHA treatment (85%). Further, compared to glucose, about ten- and four-times higher concentrations of 2-DG are required for half-maximal maintenance of GSH levels during incubation with 2 mM DHA (Figure 3D) and half-maximal inhibition of GSSG efflux (Figure 4D), respectively. Moreover, at about 400 μM intracellular GSSG, efflux rates were nearly maximal in cells supplemented with 75 μM glucose but only half maximal in the presence of 1 mM 2-DG (Figure 6B). In this context, it is interesting to note that 2-DG was described as a substrate for regeneration of GSH from GSSG in human erythrocytes, presumably by producing NADPH in the glucose-6-phosphate dehydrogenase-mediated first step of the pentose phosphate pathway (Suzuki et al., 1983). This is in line with our findings, as 2-DG partially rescued DHA-induced GSH depletion (Figure 3D). However, DHA depleted cells supplemented with 2-DG likely could not produce ATP since 2-DG blocks glycolysis, the only energy producing pathway available to erythrocytes (Pajak et al., 2019). Since MRP1/4 activity is ATP-dependent, ATP would become rate limiting in 2-DG treated cells, thus accounting for the higher cellular GSSG concentrations needed to reach half-maximal GSSG efflux rates (Figure 6B).

GLUT1-mediated DHA uptake increases the intracellular concentration of AA in erythrocytes. The elevation of cytoplasmic AA was highlighted in this study, albeit indirectly, by the reduction of intracellular ROS levels (Figure 2) and increase in PMET activity (Figure 1). The experiments were designed to best explore the capacity of DHA uptake, AA recycling and PMET activity using up to 2 mM DHA, concentrations likely not reached *in vivo*. We show that GSH depletion and GSSG efflux upon maximal DHA uptake is completely rescued even by low glucose levels, thereby indicating that erythrocytes are metabolically adapted for efficient intracellular AA recycling (Figures 3, 4). In order to put our findings in a physiological context, we will now envisage situations where GLUT1-mediated DHA uptake and increase in PMET activity may be of relevance *in vivo*. These considerations suggest that erythrocytes play an important role in systemic vitamin C metabolism.

The normal concentrations of AA in erythrocytes and blood plasma are similar and considerably low (50–80 μM) (Rumsey and Levine, 1998; Lindblad et al., 2013). However, upon oral uptake, peak AA plasma levels can rise up to 200 μM (Padayatty et al., 2004). Moreover, plasma AA levels are mostly assessed in blood sampled from antecubital veins but likely fluctuate depending on the localization within the circulatory system. Conceivably, higher AA/DHA plasma levels are encountered in vessels along the peritubular capillaries of the kidney, where AA/DHA reabsorption takes place, and along the intestinal epithelium upon food consumption (Rumsey and Levine, 1998). Epithelial cells in the intestine express GLUT1 both at the apical and basolateral membrane (Lindblad et al., 2013) thereby (i) allowing the uptake of DHA (oxidized from dietary AA by digestive processes) and (ii) partly bypassing it *via* facilitated diffusion to the blood stream, thus, promoting uptake and intracellular recycling by erythrocytes. As erythrocytes lack sodium-dependent vitamin C transporters (Nualart et al., 2014) DHA uptake does not result in persistently elevated intracellular AA levels - as known e.g., for neutrophils which have intracellular AA levels in the millimolar range (Wang et al., 1997; Bozonet and Carr, 2019). Rather, AA concentrations tend to equilibrate between erythrocytes and blood plasma either by anionic exchange of ascorbate ions or by passive diffusion of the uncharged weak acid AA (Przybylo and Langner, 2020). Thus, erythrocytes are capable of elevating AA levels in the plasma by intracellular reduction of DHA upon dietary uptake and tubular reabsorption.

Temporarily elevated AA/DHA levels are further expected in the adrenal veins, since stimulation of the adrenal glands by adrenocorticotropic hormone (ACTH) triggers the release of AA from the adrenal cortex (Padayatty et al., 2007). AA is a cofactor for the conversion of dopamine to noradrenalin (norepinephrine) by dopamine-β-hydroxylase (DβH) which takes place in chromaffin cells of the adrenal medulla. Interestingly, this process is located in the lumen of chromaffin vesicles and driven by DCytb which reduces the generated DHA to AA (Levine, 1986; van den Berg et al., 2018; Shibao et al., 2020). Intraluminal recycling of AA is dependent on cytoplasmic AA, thereby strongly resembling the PMET mechanism of and extracellular AA recycling by erythrocytes. Since adrenal catecholamines are released into the blood stream together with soluble DβH, it is tempting to speculate that erythrocytes also contribute to the process of noradrenaline generation in the adrenal veins upon activation by ACTH.

**FIGURE 7:**
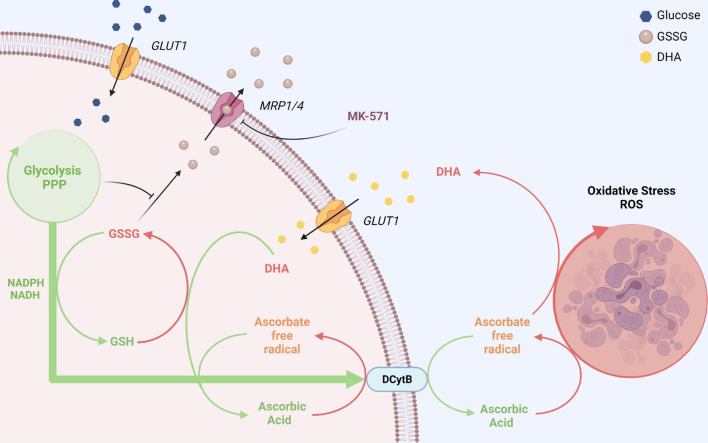
GLUT1 and DCytb-mediated vitamin C recycling as a common feature of erythrocytes from vitamin C auxotroph mammals? The plasma membrane of human erythrocytes contains high amounts of GLUT1 and DCytb. The GLUT1 glucose transporter not only facilitates the uptake of glucose but also that of DHA, the oxidized form of vitamin C. Fast intracellular reduction of DHA to AA is ensured by high cellular GSH concentrations and the high abundance of the DHA reductase GSTO-1. Thus, spontaneous decomposition and irreversible loss of the labile DHA is prevented. DHA-dependent generation of GSSG and its export *via* MRP1/4 is prevented in the presence of physiological glucose concentrations. Furthermore, increased intracellular AA levels reduce intracellular ROS levels (not depicted here) and fuel DCytb-mediated plasma membrane electron transport (PMET). While in duodenal enterocytes DCytb mediates Fe^3+^ reduction for dietary iron absorption, the main extracellular substrate of DCytb in the erythrocyte membrane appears to be ascorbate free radical (AFR), which is consequently reduced to AA. Both processes, (i) uptake of DHA and its intracellular reduction, and (ii) DCytb-mediated PMET activity which recycles extracellular AA from AFR (generated upon reduction of ROS in the plasma), can be considered a “vitamin C recycling mechanism.” This underestimated physiological function of human erythrocytes likely contributes to (i) oxidative stress defense in blood and (ii) sustainability of systemic AA supply, considering the fluctuating dietary uptake. The importance of this system is underlined by the finding that, in contrast to most other mammalian species, erythrocytes of vitamin C auxotroph mammals harbor GLUT1 and possibly also DCytb (see section “Discussion”). “Created with BioRender.com”.

DHA uptake and PMET activity of erythrocytes may further play an important role in oxidative stress response. Oxidative stressors and ROS in the blood plasma are efficiently detoxified by AA (Frei et al., 1989) at the cost of AFR and DHA generation. DHA is efficiently absorbed by erythrocytes and immediately reduced to AA, a process enabled by high intracellular GSH levels. The transient accumulation of AA in erythrocytes in turn strongly enhances PMET activity which largely potentiates extracellular AA regeneration by AFR reduction *via* DCytb (Figure 1A). Physiological blood glucose levels (i) are sufficient to continuously restore the full reductive capacity of erythrocytes by regenerating intracellular GSH and concomitantly preventing GSSG accumulation as well as efflux (Figures 3–6) and (ii) drive high capacity, long lasting PMET activity for extracellular ROS defense (Figures 1B, 7).

In short, erythrocytes prevent loss of dietary AA by rapid uptake of DHA and extracellular reduction of AFR - processes mediated by GLUT-1 and DCytb, respectively. While high level expression of GLUT-1 has already been recognized as an (most likely) essential adaptation in vitamin C auxotroph mammals (Montel-Hagen et al., 2008; Hornung and Biesalski, 2019) a similar impact of DCytb has not been considered yet. Indeed, DCytb is abundant in erythrocytes of *homo sapiens* and guinea pigs but absent in erythrocytes of mice and rats, two rodent species capable of AA-biosynthesis (Su et al., 2006). This raises the thrilling question whether expression of DCytb at the erythrocyte membrane is a second necessary adaptation to compensate for the loss of AA biosynthesis. Corresponding analyses of erythrocytes of higher apes and fruit bats will tell and may contribute to our understanding of molecular mechanisms at work during evolution to select for and adapt to novel traits. From a systemic perspective, “outsourcing” AA production can even be viewed as beneficial, since AA biosynthesis produces the oxidant H_2_O_2_ as a by-product in the final GLO-mediated step of synthesis. Obviously, this outsourcing requires a permanent AA supply which must be sufficient to reduce the selection pressure for AA biosynthesis (Drouin et al., 2011). However, in view of the amazing coincidence of GLUT1 (and possibly also DCytb) expression in erythrocytes and vitamin C auxotrophy (in the respective species), it is tempting to assume that these changes in erythrocyte redox properties were a prerequisite for the loss of GLO activity without causing an evolutionary disadvantage. Conceivably, erythrocyte-mediated vitamin C recycling, both intracellular *via* GLUT1-mediated DHA uptake and reduction as well as extracellular *via* DCytb-mediated AFR reduction, contributes to a “sustainable” usage of this antioxidant by minimizing its loss in the blood stream and boosting its efficacy *via* PMET activity. Improvement in sustainable vitamin C usage by shifting to erythrocytes expressing high levels of GLUT1 and DCytb may indeed have been prerequisite steps ahead of the emergence of vitamin C auxotrophy. Only after that had been established, selection pressure on maintenance of endogenous AA biosynthesis was gone and inactivation of the GLO gene could take place. Interestingly, this evolutionary selection process would have independently occurred at least twice in the phylogeny of mammals (Drouin et al., 2011).

## Data Availability Statement

The raw data supporting the conclusions of this article will be made available by the authors, without undue reservation.

## Ethics Statement

The studies involving human participants were reviewed and approved by Ethics Commission of the Medical University of Vienna (EK Nr. 1752/2020). The patients/participants provided their written informed consent to participate in this study.

## Author Contributions

ME and US designed the study and drafted the manuscript. ME, DS, CZ, and SG acquired the data. ME, DS, CZ, SG, MF, DB, EM, and US analyzed and contributed to the interpretation of the data. EM and DB revised the manuscript critically. All authors approved the final version.

## Conflict of Interest

The authors declare that the research was conducted in the absence of any commercial or financial relationships that could be construed as a potential conflict of interest.

## Publisher’s Note

All claims expressed in this article are solely those of the authors and do not necessarily represent those of their affiliated organizations, or those of the publisher, the editors and the reviewers. Any product that may be evaluated in this article, or claim that may be made by its manufacturer, is not guaranteed or endorsed by the publisher.
